# Terrestrial vegetation and aquatic chemistry influence larval mosquito abundance in catch basins, Chicago, USA

**DOI:** 10.1186/1756-3305-6-9

**Published:** 2013-01-11

**Authors:** Allison M Gardner, Tavis K Anderson, Gabriel L Hamer, Dana E Johnson, Kate E Varela, Edward D Walker, Marilyn O Ruiz

**Affiliations:** 1 Department of Pathobiology, University of Illinois at Urbana-Champaign, 2001 South Lincoln Avenue, Urbana, IL, 61802, USA; 2 Virus and Prion Research Unit, National Animal Disease Center, USDA-ARS, Ames, IA, 50010, USA; 3 Department of Entomology, Texas A&M University, TAMU 2475, College Station, TX, 77843, USA; 4 Department of Microbiology and Molecular Genetics, Michigan State University, 2215 Biomedical Physical Sciences, East Lansing, MI, 48824, USA

**Keywords:** *Culex* mosquitoes, Larval habitat, Landscape ecology, Vegetation, Aquatic chemistry, Geographic information science, West Nile virus

## Abstract

**Background:**

An important determinant of mosquito-borne pathogen transmission is the spatial distribution of vectors. The primary vectors of West Nile virus (WNV) in Illinois are *Culex pipiens* Linnaeus (Diptera: Culicidae) and *Culex restuans* Theobald. In urban environments, these mosquitoes commonly oviposit in roadside storm water catch basins. However, use of this habitat is inconsistent, with abundance of larvae varying significantly across catch basins at a fine spatial scale.

**Methods:**

We tested the hypothesis that attributes of the biotic and abiotic environment contribute to spatial and temporal variation in production of mosquito vectors, characterizing the relationship between terrestrial vegetation and aquatic chemistry and *Culex* abundance in Chicago, Illinois. Larvae were sampled from 60 catch basins from June 14 to October 3, 2009. Density of shrubs and 14 tree genera surrounding the basins were quantified, as well as aquatic chemistry content of each basin.

**Results:**

We demonstrate that the spatial pattern of *Culex* abundance in catch basins is strongly influenced by environmental characteristics, resulting in significant variation across the urban landscape. Using regression and machine learning techniques, we described landscape features and microhabitat characteristics of four Chicago neighborhoods and examined the implications of these measures for larval abundance in adjacent catch basins. The important positive predictors of high larval abundance were aquatic ammonia, nitrates, and area of shrubs of height <1 m surrounding the catch basins, whereas pH and area of flowering shrub were negatively correlated with larval abundance. Tree density, particularly of arborvitae, maple, and pear, also positively influenced the distribution of *Culex* during the fruit-bearing periods and early senescent periods in August and September.

**Conclusions:**

This study identifies environmental predictors of mosquito production in urban environments. Because an abundance of adult *Culex* is integral to efficient WNV transmission and mosquitoes are found in especially high densities near larval habitats, identifying aquatic sites for *Culex* and landscape features that promote larval production are important in predicting the spatial pattern of cases of human and veterinary illness. Thus, these data enable accurate assessment of regions at risk for exposure to WNV and aid in the prevention of vector-borne disease transmission.

## Background

Heterogeneous patterns of infection at various spatial scales are evident in many zoonotic disease systems, including mosquito-borne viruses, such as West Nile virus (WNV) [1,2], and those vectored by other arthropods [3,4]. This variation in disease distribution is to be expected, because biotic and abiotic habitat characteristics differ between locations and these distinctions can either facilitate or inhibit the establishment and persistence of pathogens [5]. Environmental features may influence viral risk of human illness by altering the transmission competence and fitness of vectors, the infection prevalence in the vertebrate reservoir host population, and the abundance and spatial distribution of all three organisms involved in the arboviral transmission cycle: hosts, parasites, and vectors. In WNV, for example, high ambient temperature increases the mosquito development rate [6] and pathogen transmission capacity [7,8], decreases the viral incubation period [9], and alters amplification processes in the virus’ avian reservoir hosts (in North America, these include the American robin, *Turdus migratorius*, and the house sparrow, *Passer domesticus*) [10]. Human modification of habitat, such as introduction of pesticides, fertilizers, and xenobiotics to the aquatic larval environment, has also been shown to influence the vector competence of adult mosquitoes, affecting the development of mosquitoes and altering the outcome of inter- and intra-specific competition among larvae [11,12].

An important driver of enzootic transmission in mosquito-borne viruses is the abundance and distribution of the vector community. *Culex pipiens* Linnaeus (Diptera: Culicidae) and *Culex restuans* Theobald (hereafter “*Culex*”) are the most common amplification vectors for WNV in the east north central United States [13], and are also bridge vectors of WNV to humans, equines, and other mammalian ancillary hosts [14]. These container breeders are generalists in aquatic habitat use, with larvae and pupae found in both natural and artificial habitats: gutters, metal and plastic containers, discarded tires, bird baths, rain barrels, vernal ponds, and stagnant pools of water [15,16]. Roadside storm water catch basins have also been identified as important oviposition sites for *Culex* mosquitoes in urban locales [17,18].

The abundance and infection prevalence of adult *Culex* mosquitoes have been linked to landscape features including vegetation cover [19], demographic characteristics and structure of residential neighborhoods [20], and spatial and temporal weather patterns [21]. However, fewer studies have explored the relationship between landscape characteristics of urban neighborhoods and distribution of *Culex* larvae. These data are important for two reasons. First, laboratory studies have found that many physiological traits that later drive the effectiveness of adults as WNV vectors develop as the direct products of environmental exposures in the larval stage [22]. Second, abatement efforts are centered on the identification of important oviposition habitats and control of mosquitoes in the aquatic immature stages. Therefore, it is of interest to public health districts and others responsible for mosquito abatement to understand natural sources of variation in vector production, and apply this knowledge to disrupt the ecological pathways that favor high vector abundance and concomitant elevated pathogen transmission.

Prior research has revealed that weather is one of the most important predictors of *Culex* larval abundance in the storm water catch basin system, explaining much of the temporal variation in urban larval production. Large (>3.50 cm) multi-hour rainfall events within four days preceding collection have been shown to “flush” almost all mosquito larvae from underground storm drains and to limit adult production in catch basins for up to a week in metropolitan Chicago [22]. High ambient and aquatic temperatures have also been shown to accelerate larval production and development rates [6]. These effects do not influence all catch basins equally: some catch basins are protected from heat and rainfall by overhanging trees, and catch basins surrounded by impervious surfaces are more susceptible to street chemical and fertilizer runoff due to high rainfall than those surrounded by grass. However, weather generally is a broad scale exposure, and though it may contribute significantly to temporal variation in *Culex* production, it likely is not a key determinant of the inconsistent use of catch basin habitats within a narrow geographic range.

The purpose of this study is to examine sources of fine-scale environmental heterogeneity in an aquatic container habitat that influence *Culex* larval production, and help explain why two catch basins in close proximity may vary dramatically in larval abundance. One landscape feature that largely has been overlooked in relation to *Culex* abundance in field studies is terrestrial vegetation genus and structure. Laboratory studies have demonstrated that the substrates of different generas of tree leaves in the aquatic environment alter adult survival, development rate, and longevity, as well as outcomes of inter- and intra-specific competition due to differential toxicity of detritus genera to mosquito larvae and potential differences in the effects of plants on the aquatic chemistry of the larval habitat [23,24]. However, to the authors’ knowledge no previous research has investigated the influence of surrounding vegetation on *Culex* production in the epidemiologically relevant man-made habitats provided by storm water catch basins.

Trees and shrubs may impact the spatial pattern and abundance of *Culex* larvae at both broad and fine scales. At the neighborhood level, dominant street tree genus may have important implications for larval production; for instance, areas with high oak densities may produce fewer mosquitoes because the tannins contained in their leaves are toxic to the aquatic stages [25]. On a local scale, trees and shrubs may determine the distribution of mosquitoes due to their importance as sugar feeding sources for adult mosquitoes as well as resting sites for both adult mosquitoes and their avian blood meal hosts, encouraging gravid females to oviposit in nearby catch basins and other proximate aquatic habitats. At the microhabitat level, organic detritus of vegetation may alter the aquatic chemistry in catch basins, potentially influencing the attractiveness of these habitats to ovipositing females and adult emergence rates. Further, because larvae feed on microorganisms in the water column, the algal growth promoted by nitrogen contained in leaves may support *Culex* production [26,27].

To test the hypothesis that attributes of the biotic and abiotic environment play a role in the variation observed in the spatial distribution of important vector species, we characterized the effect of vegetation and aquatic chemistry on the abundance of WNV vectors in metropolitan Chicago, Illinois. We used regression and machine learning statistical techniques to identify vegetation features and aquatic chemistry parameters associated with high abundance of *Culex* larvae. We focused our analysis on density of 14 genera of common trees, area of shrubs and ornamental grasses surrounding catch basins, and pH, nitrate, phosphate, and ammonia content of catch basin water.

## Methods

### Larval abundance

Sampling was conducted in four residential municipalities in metropolitan Chicago, Illinois (Cook County; 87’44” W, 41’42” N). Evergreen Park is a suburban village with an area of 8.2 km^2^ and a population of 19,237 in 2009. Alsip has an area of 16.5 km^2^ and a population of 18,580. Oak Lawn (subdivided into north and south regions) has an area of 22.3 km^2^ and a population of 52,948. WNV became established in the region during the summer of 2002 with 884 human cases state-wide [28], at the time the largest reported WN meningoencephalitis epidemic. We selected the study area for its notable concentration of virus-positive mosquitoes and birds and cases of human illness over the past eight years [21,29], as well as its diverse composition of street trees and shrubs in residential neighborhoods [30].

**Figure 1:**
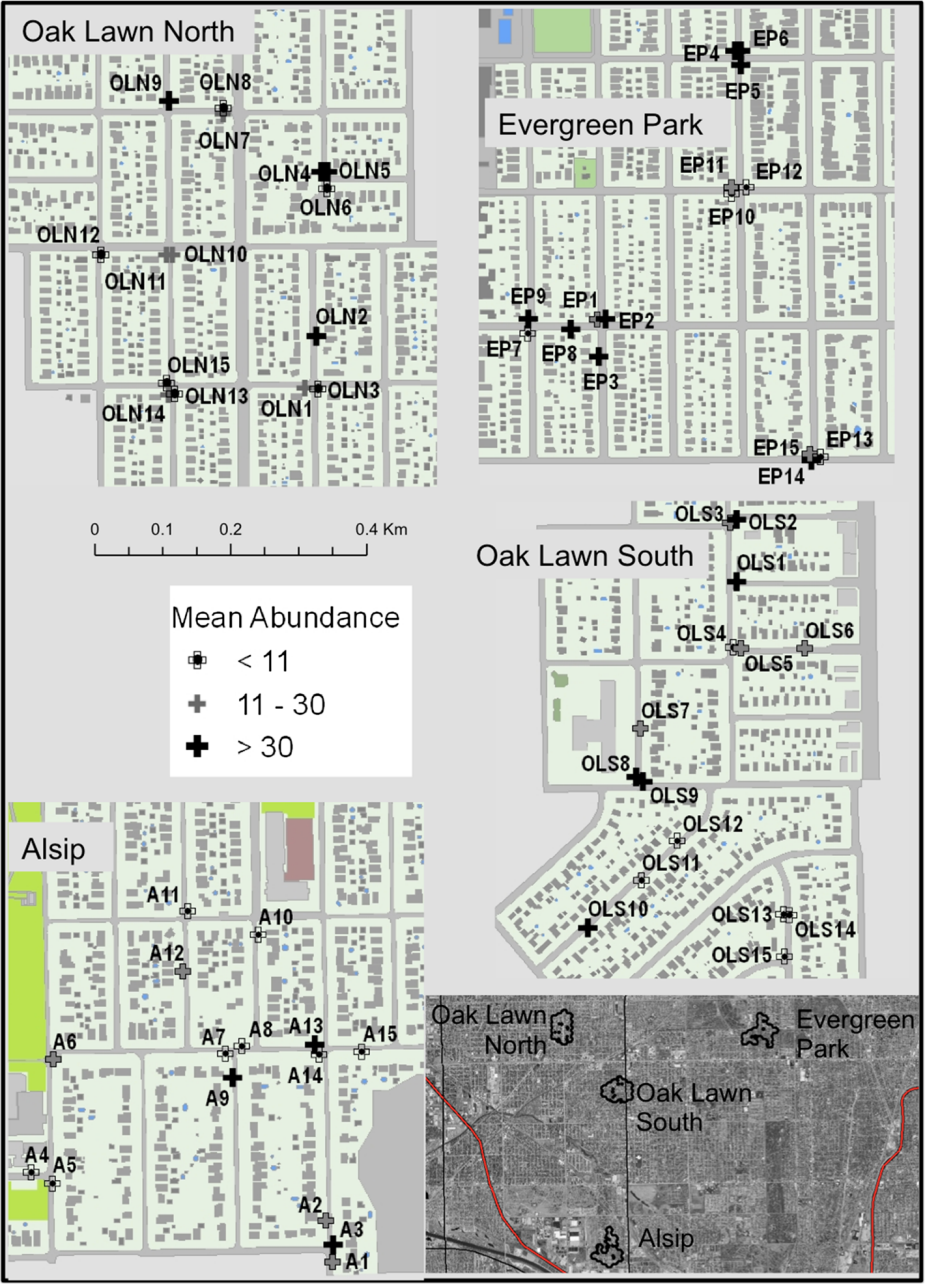
**Sixty larval sampling catch basins in four metropolitan Chicago neighborhoods (Alsip, Evergreen Park, Oak Lawn North, and Oak Lawn South).** Black-and-white crosses indicate low larval abundance (average <11 larvae per sample), grey crosses indicate medium abundance (average 11–30 larvae per sample), and black crosses indicate high larval abundance (average >30 larvae per sample).

To estimate the mean abundance of mosquito larvae per catch basin, we sampled 60 catch basins: 15 basins within each of four neighborhoods (named here as Evergreen Park, Alsip, Oak Lawn North, and Oak Lawn South) (Figure 1). The mean depth from street surface to the bottom of the basin was 68 ± 20.22 cm with a diameter of 60 cm. All basins had open grates and were located on the edges of residential suburban streets. The basins were sampled for larvae once per week from June 14 to October 3, 2009 according to methods described in Hamer *et al.* (2011) [31]. Larvae and pupae were collected using a 10.2 X 10.2 cm aquarium net attached to the end of a conduit pole, 3 m in length and 1.3 cm in diameter. The pole was inserted through the grate and passed over the water surface in a single figure eight. The net was then inverted into a container and all larvae were collected, counted, and identified to species using taxonomic keys [32].

**Figure 2:**
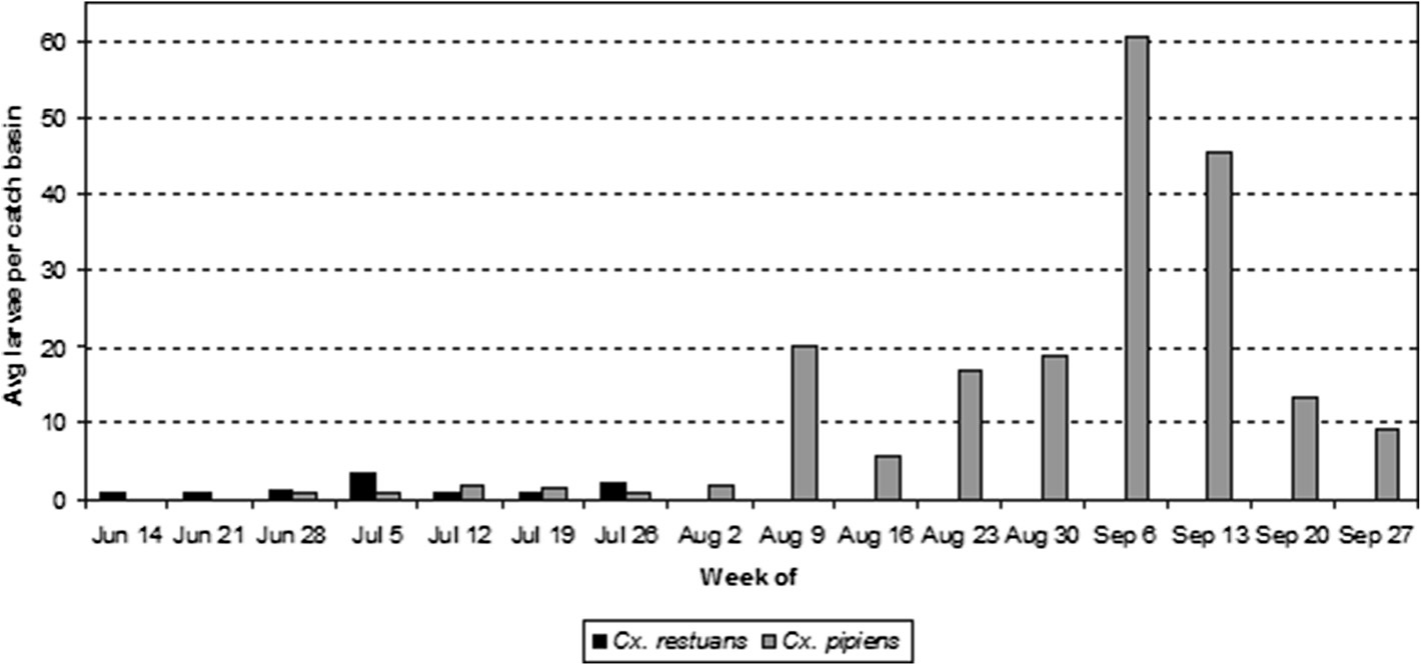
**Seasonal abundance of *Culex pipiens *and *Cx. restuans *throughout the study period from the week of June 14th to September 27th, 2009.**

Larval abundance of *Culex pipiens* and *Culex restuans* were aggregated for all statistical analysis. This is an appropriate approach because larvae of both species often share similar habitats [18], feed on similar hosts [33], and function similarly as enzootic disease vectors [34]. In our study region, *Cx. restuans* and *Cx. pipiens* occur at comparable abundances early in the summer and by mid-summer, *Cx. pipiens* dominates (Figure 2) [35].

### Vegetation

To characterize the vegetation types and structures surrounding the catch basins, trees and shrubs in the four study neighborhoods were mapped using methods modified from the U.S. Forest Service Urban Forest Project [36]. On-site surveys were conducted with boundaries surrounding catch basins by 25 m. Only trees greater than 3 m in height were identified to genus. A small proportion of plants in this zone were unidentifiable to the surveyor or were not visible from the street. They were classified as “unknown” or “obscured” respectively. Tree densities within 25 m of each basin were calculated using kernel density estimation in ArcGIS 10 (ESRI Inc., Redlands, CA) with a cell size of 10 m^2^, a search radius of 100 m, and Silverman’s rule of thumb for bandwidth selection [37]. Shrub area was quantified within 25 m of each basin. Shrubs were classified by foliage type (evergreen, deciduous, flowering, or ornamental grass) and height (<1 m, 1–2 m, or 2–3 m).

### Aquatic chemistry

To quantify the aquatic chemistry of the catch basins, water quality parameters were tested by calorimetric analysis using Chemetrics CHEMets test kits (Chemtech International Inc., Irvine, CA). We tested for ammonia (Kit K-1510, direct nesslerization method), phosphate (K-8510, stannous chloride chemistry), and nitrate (K-6904, cadmium reduction method). Basin pH was measured using the Chemetrics double junction pH meter (Cat. No. I-1000). To quantify the broad seasonal trends in water quality observed in other stream and stagnant water ecosystems [38,39], these parameters were measured twice during the study period for all basins (weeks of June 28 and August 16). To capture fine-scale temporal variation in water quality, the same parameters were measured weekly for eight basins spaced throughout the study area. Water samples were obtained by attaching an aquarium net frame to the end of a conduit pole, 3 m in length and 1.3 cm in diameter. A plastic sealable bag was secured to the frame with foldback clips. The pole was inserted through the grate and submerged under water until the bag was at least half full. Aquatic pH was measured immediately. The sample was then stored in a cooler on ice for no more than 6 hours prior to processing.

### Statistical analysis

The following environmental metrics were examined in relation to *Culex* larval abundance: area of shrubs of height <1 m, 1–2 m, and 2–3 m within 25 m of each catch basin; area of shrubs of foliage types deciduous, evergreen, flowering, and ornamental grass within 25 m of each basin; density of deciduous trees and evergreen trees within 25 m of each basin; and pH, ammonia, phosphate, and nitrate content of each basin. To determine which independent variables were correlated to larval abundance, we conducted forward stepwise regression in SAS 9.2 (SAS Institute Inc., Cary, NC). The significance level α = 0.05 was required for entry into the model. The dependent variable was average mosquito abundance for each basin. To quantify the temporal structure of larval abundance, this analysis was conducted for early season and late season time windows. The early season window was based on larval abundance for June and July and the aquatic chemistry measurements obtained on June 28th, 2009 and the late season window was based on larval abundance for August and September and the aquatic chemistry measurements were obtained on August 16th. Terrestrial vegetation was assumed to remain constant throughout both time groups.

To examine which tree genera were most closely related to larval *Culex* abundance, we conducted regression tree (RT) and random forest (RF) nonlinear regression using the packages rpart [40] and randomForest [41] in R 2.12.1 (R Foundation for Statistical Computing, Vienna, Austria). The most commonly observed local tree genera were considered in this procedure. Deciduous trees included ash (*Fraxinus* spp.), birch (*Betula* spp.), cottonwood (*Populus* spp.), crabapple (*Malus* spp.), elm (*Ulmus* spp.), locust (*Gleditsia* spp.), magnolia (*Magnolia* spp.), maple (*Acer* spp.), oak (*Quercus* spp.), pear (*Pyrus* spp.), and plum (*Prunus* spp.). Evergreen trees included arborvitae (*Thuja* spp.), pine (*Pinus* spp.), and spruce (*Picea* spp.). All of these genera were observed more than 50 times within the study area. Again, to examine the variable effect of tree genera throughout the season, the analysis was repeated for the early season and late season time windows.

RT and RF belong to the Classification and Regression Tree (CART) family of nonparametric decision tree models described by Breiman *et al.* (1984) [42]. In RT, the variation in the response variable (mean larval abundance per catch basin) is recursively partitioned along binary nodes of predictive covariates (density of each tree species), maximizing the homogeneity within each partition. The resulting “tree” of nested covariates demonstrates the relative amount of variation in the response explained by each predictor. RF is a bootstrapping method based on iterations of RT, in which both predictors and responses are randomly permuted. The robustness of the classifications determined by RT is assessed based on the total decrease in node impurities from splitting the variable, measured by residual sum of squares. Tree-based modeling handles missing covariates, may combine quantitative and qualitative covariates, and does not have the assumptions of generalized linear mixed models and neural networks, among other alternatives for quantitative data [43]. These procedures have been used to analyze data in multiple mosquito-borne disease systems [22,44].

## Results

### Larval abundance

A total of 960 samples were collected from 60 catch basins from June 14 to October 3, 2009. The mean number of larvae per collection was 19 for the early season and 27 for the late season, with the largest larval samples taken at site IDs OLN13, A5, A11, and A15 and the smallest samples taken at site OLN5 (Figure 1). *Culex restuans* was the dominant species in the early season, comprising 65 percent of all larvae collected during that time window (n = 831). *Culex pipiens* was the only species collected during the late season window (n = 10,770) (Figure 2). There were no non-*Culex* mosquitoes recorded in catch basin samples throughout the period. Other invertebrates including some potential mosquito predators such as copepods were collected in the basins, but these occurred in low abundance and were not tabulated.

### Vegetation and aquatic chemistry

**Figure 3:**
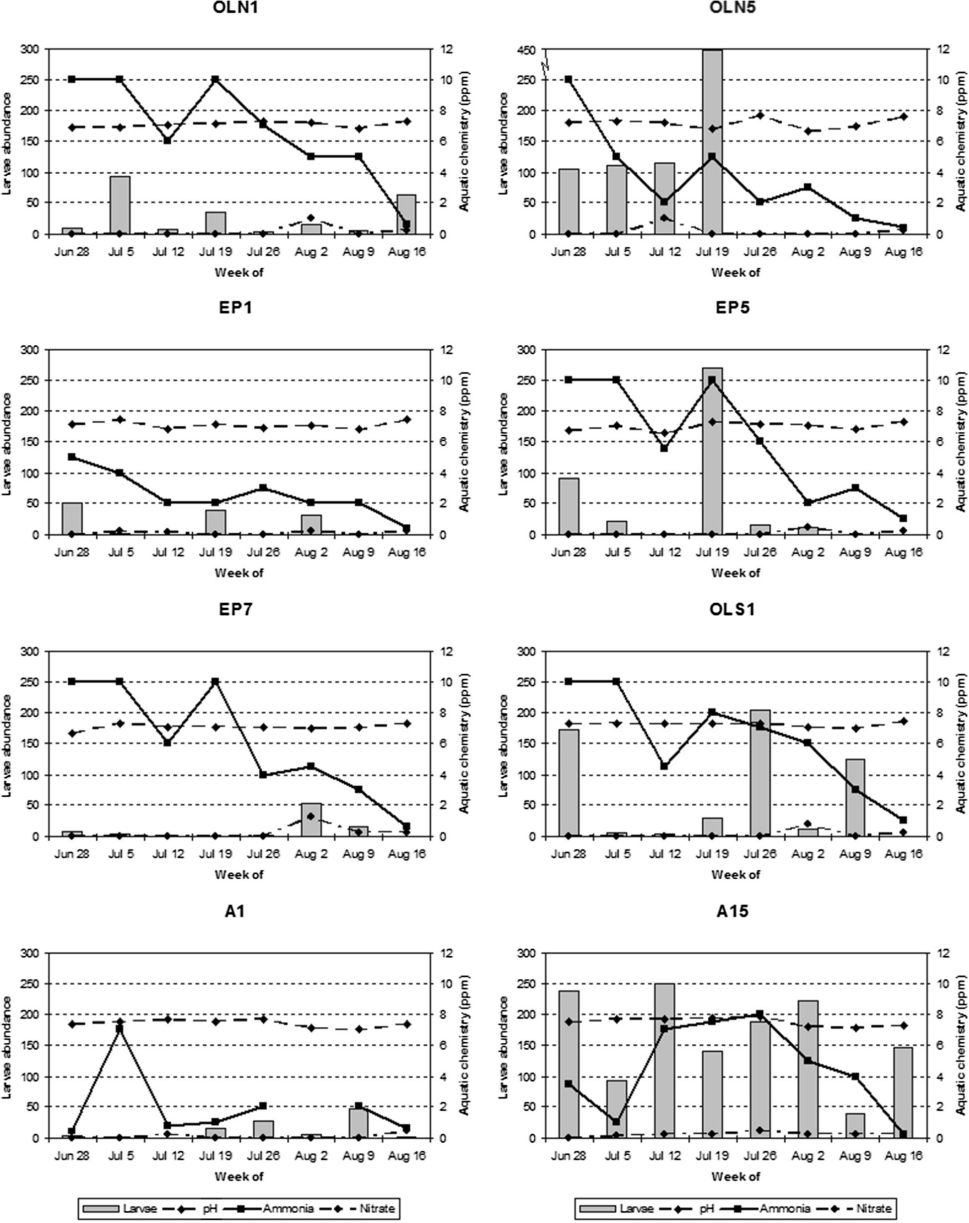
Larval abundance and aquatic chemistry content (pH, ammonia, and nitrate) for eight catch basins sampled weekly from June 28th to August 16th, 2009.

Forward stepwise linear regression fitted significant models describing variation in *Culex* larval abundance among the 60 catch basins for the early (n = 57; R^2^ = 0.24; p < 0.001) and late season (n = 58; R^2^ = 0.31; p = 0.003) time windows. The retained variable for the early season model was aquatic ammonia, indicating that ammonia is positively correlated to larval abundance. The retained variables for the late season model were aquatic pH, ammonia, and nitrate, area of flowering shrubs and shrubs <1 m height, and density of deciduous trees within 25 m of the basin. Of these, aquatic ammonia, aquatic nitrate, and short shrub area were positively correlated to larval abundance while aquatic pH and flowering shrub area were negatively correlated to larval abundance (Table 1). Among the eight basins sampled weekly for aquatic chemistry, there was a comparable temporal association between larval abundance and pH, ammonia, and nitrate content (Figure 3).

**Table 1:** **Multiple forward stepwise regression model of *Culex *larvae in relation to terrestrial vegetation and aquatic chemistry characteristics for early season (June-July) and late season (August-September) time windows**

**Parameter**	**Coefficient**	**Standard Error**	**Std. Coefficient**	**T**	**P**
Early season (June-July)					
Ammonia	5.51	1.33	0.49	4.16	<0.001*
Late season (August-September)					
Ammonia	25.25	8.55	0.37	2.96	0.005*
pH	−14.74	8.60	−0.21	−1.71	0.093^†^
Nitrate	16.43	8.61	0.26	1.91	0.062^†^
Flowering shrubs	−1.40	0.70	−0.36	−2.01	0.050*
Shrubs height <1 m	0.67	0.46	0.29	1.45	0.152^†^
Deciduous trees	1.69	1.41	0.14	1.19	0.238

*Indicates a statistically significant result, α = 0.05.

†Indicates a marginally significant result, α = 0.20.

### Tree genera

Regression tree (RT) and random forest (RF) nonlinear regression fitted models for larval abundance as a function of density of 14 tree genera within 25 m of each catch basin in the early season and late season time windows. The RT models outperformed the RF models for both early season (R^2^ = 14.4) and late season (R^2^ = 45.6). The RF model was a poor fit for early season data (pseudo-R^2^ = −23.1), although the same model demonstrated substantially stronger predictive power for late season data (pseudo-R^2^ = 42.0).

**Figure 4:**
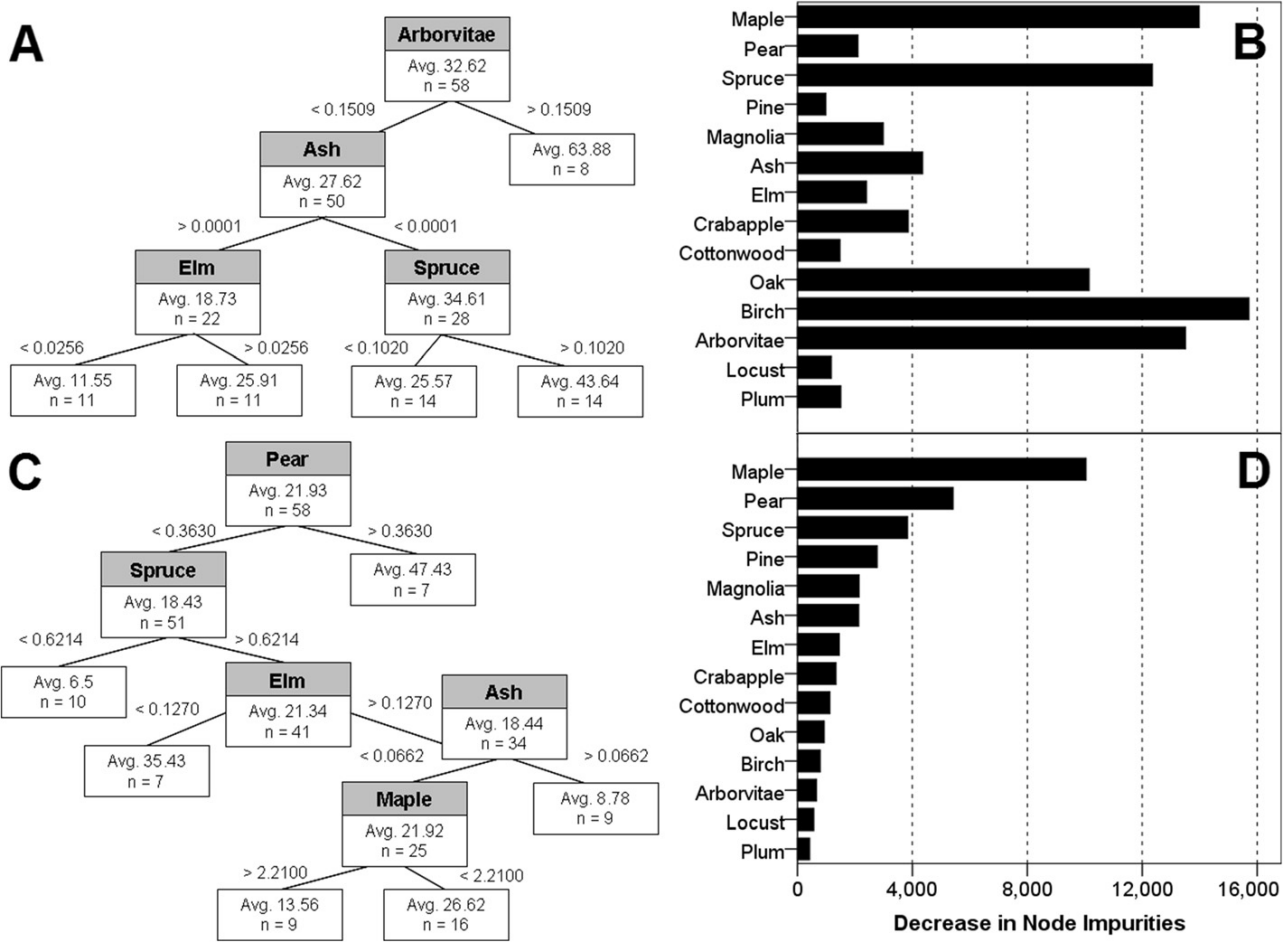
Regression tree models (A, C) and variable importance scores for random forest models (B, D) of tree genera density as predictors of larval abundance in the early season (A, B) and late season (C, D) time windows.

The early season RT model showed arborvitae, spruce, and elm tree density positively associated with *Culex* larval abundance and ash tree density negatively associated with larval abundance (Figure 4A). These results were verified by the RF model, which suggested that spruce and arborvitae, in addition to maple, birch, and oak, were most strongly associated with larval abundance (Figure 4B). The late season RT model showed pear, spruce, and elm tree density positively correlated to larval abundance and ash and maple tree density negatively correlated (Figure 4C). Again, these results were supported by the RF model, which showed these genera and pine and magnolia as the most important predictors of larval abundance (Figure 4D).

## Discussion

Our results demonstrate that *Culex* larval abundance varies spatially and temporally across urban landscapes and is strongly influenced by environmental characteristics, including the surrounding vegetation structure and aquatic chemistry. Using regression and machine learning techniques, we described the landscape features and fine-scale microhabitat characteristics of four Chicago neighborhoods and examined the implications of these measures for larval abundance in adjacent catch basins. We determined that aquatic pH, ammonia, and nitrates, terrestrial deciduous and flowering shrub area, and tree density are predictors of larval production in catch basins. However, the relative importance of these effects varies temporally, with aquatic chemistry influential in the early season and vegetation increasing in significance in the late season. These data may be used to inform mosquito control efforts by demonstrating the consequences of landscaping decisions on local mosquito production, and subsequently aid in the reduction of human risk of exposure to mosquito-borne disease in residential neighborhoods.

Potential effects of vegetation on larval abundance generally fall into two categories: First, trees and shrubs may offer resting habitats and sugar sources to adult females and their avian blood meal hosts, encouraging the mosquitoes to oviposit in nearby catch basins. Second, trees and shrubs may provide direct detrital inputs to the catch basins. Leaf litter and fallen fruit may affect mosquito production by influencing aquatic chemistry, introducing biomolecules to the larval habitat, and altering abundance and composition of the microbial community within the catch basin. Collectively, these effects have the potential to alter the attractiveness of the aquatic habitat to gravid females as well as the quality of the aquatic habitat for developing larvae.

Because there is often a positive correlation between abundance of adult mosquitoes and abundance of larvae in urban storm water systems [45,46], shrubs may have an important role in determining larval production in catch basins by affecting the abundance of gravid females nearby. We found a marginally significant positive association between larval abundance and area of shrubs <1 m height within 25 m of each catch basin. In contrast, area of flowering plants was negatively associated with larval abundance. A possible explanation for this pattern is that male and female mosquitoes as well as potential avian blood meal hosts are attracted to non-flowering shrubs as resting sites and sources of fruit, encouraging gravid females to oviposit in catch basins and other aquatic habitats proximate to the site of their blood meals. Conversely, flowering plants containing pyrethroid compounds (*e.g.*, chrysanthemum) and natural volatile oils (*e.g.*, geranium, peppermint, and rosemary) may repel adult mosquitoes, and thus reduce the number of adults present and potential for oviposition in nearby catch basins [47,48].

This relationship between vegetation proximate to catch basins and larval production was observed in trees as well as shrubs. Although linear regression indicated that total tree density was not correlated to larval abundance, this finding did not reflect broadly posed unimportance of trees in determining *Culex* production, but rather the sum of non-uniform effects of different genera on larval abundance. Regression tree and random forest models showed that key tree genera may have significant positive or negative effects on larval production. For example, in the early season, arborvitae and spruce trees were positively associated with larval abundance, suggesting that these shrubby, low-hanging trees serve a similar ecological function to shrubs in providing a resting and feeding site for *Culex* females and their avian hosts. The increased importance of pear trees to larval abundance during the fruit-bearing period in the late season indicates that trees not only provide habitat to adult mosquitoes, but may also offer a possible sugar source for females and males and an attractant to blood meal hosts [49]. Few other fruiting trees were observed in the study area in sufficiently high densities for consideration in our models, and the effect of plums, mulberries, and other fruit-bearing trees on mosquito diet and host presence remains open to future study.

Vegetation also provides a direct source of detritus to nearby catch basins, with several potential implications for larval abundance. First, vegetation has been demonstrated to alter the aquatic chemistry of larval environments in naturally occurring and artificial container habitats, such as tree holes and used tires [50], and our findings in the catch basin ecosystem were consistent with these previous results. We found that larval abundance in catch basins was positively correlated with broad, seasonal trends in aquatic nitrate and ammonia variation. The organic detritus of plants introduced throughout the year, including pollen, flowers, fruit, seeds, and leaves, likely is a primary source of nitrogen in catch basins, explaining an increase in nitrates in catch basins during the late season after a critical mass of trees had dropped fruit and entered senescence [26,27]. Elm trees, positively correlated to larval abundance, may have contributed substantially to this effect in our study area because their leaves lack a thick wax coating and therefore decompose quickly in water.

The impact of natural inputs on aquatic chemistry in the urban environment also may be compounded by artificial inputs and xenobiotics, such as lawn fertilizers, road salt, pesticides, and herbicides. Stemflow has been shown to dilute the chemical concentration of treehole microhabitats [51], and a similar effect of groundwater could explain additional variance in our models. Further, it is noteworthy that catch basins are subject to constant fluctuations in pH, ammonia, and nitrates, as we observed in the eight catch basins tested weekly for water quality concurrent with larval sampling. From our qualitative interpretation of that sample size, weekly larval abundance appears to track with these parameters. Our early season and late season models based on fewer aquatic chemistry measurements are significant because they capture the influence of broad scale, seasonal patterns in water quality [38,39] on larval production, knowledge that is important to the accurate prediction of annual and inter-annual variation in mosquito abundance. Further research could clarify the function of fine-scale temporal aquatic chemistry fluctuations in determining vector production.

Second, vegetation introduces biomolecules to catch basin aquatic communities. These compounds, including tannins, cellulose, and glucose, may have significant effects on *Culex* production. While the catch basin sample size and water quality parameters taken in our current study were insufficient for correlation analysis examining the relationship between vegetation characteristics and biomolecules, prior studies have demonstrated that the compounds contained in the leaves of certain tree species may create environments inhospitable to *Culex*[25]. For example, the tannins contained in oak leaves are toxic to the aquatic stages of mosquitoes [52], so that catch basins near these trees may have low larval abundance. Laboratory study could reveal whether detritus of ash and maple trees, such as wind-dispersed samaras, leaves, and branches, have comparable physiological effects on developing mosquitoes.

Finally, vegetation detritus may alter aquatic habitat quality and mosquito production via its effect on the microbial flora in catch basin mesocosms. It is understood that microbial abundance is related to leaf biomass in aquatic habitats [53,54], and that microbes contribute to the larval diet through nutrient cycling and by providing a direct food source to developing larvae [55,56]. However, microbial communities likely vary substantially among substrates of different leaf genera and thereby may alter mosquito production and fitness. One mechanism that may be responsible for associations between tree genera surrounding catch basins and mosquito production is differences in abundance and composition of microbial communities that grow on each leaf genus. These relationships between leaf detritus type, bacterial flora, and mosquito abundance and distribution should be examined in future investigations.

## Conclusions

An important predictor of human illness in multiple mosquito-borne disease systems [57,58] is local abundance of vectors. Studies of mosquito ecology in catch basin and underground storm drain systems [45,46] among other larval habitats [59] have demonstrated that adult mosquitoes are often found aggregated near standing water breeding environments. Because an abundance of adult *Culex* is integral to efficient WNV transmission and mosquitoes are found in especially high densities near oviposition locations, identifying breeding sites for *Culex* and assessing the landscape features that promote larval production are important in predicting the spatial pattern of cases of human disease.

The current study contributes to our knowledge of the environmental conditions that favor larval production in the urban ecosystem, thus enabling more accurate assessment of populations at risk for human illness. Further, our results may be used to guide mosquito district protocols for larval control. Although catch basins may be treated with larvicides to eliminate larvae or inhibit larval development and thus reduce adult emergence rates [60], many public health departments lack the material and human resources to sample catch basins continuously throughout the summer and treatments lose their effect over time. Focusing chemical treatment on neighborhoods with high shrub and deciduous tree densities may help target limited supplies to locations especially likely to produce vectors.

## Competing interests

The authors declare that they have no competing interests.

## Authors’ contributions

AG conceptualized and carried out the statistical analysis and was lead author in writing and revision of the manuscript. TA and GH contributed to the background literature, helped with interpretation and conclusions, and critiqued the statistical analysis. DJ and KV collected field data, including mosquito abundance, terrestrial vegetation, and aquatic chemistry data. EW conceptualized field sampling methods and contributed to interpretation and conclusions. MR defined and directed the research questions and methods, contributed to the statistical analysis, and helped to write the manuscript. All authors read and approved the final version of the manuscript.
